# Emergence of B.1.524(G) SARS-CoV-2 in Malaysia during the third COVID-19 epidemic wave

**DOI:** 10.1038/s41598-021-01223-4

**Published:** 2021-11-11

**Authors:** Kim-Kee Tan, Jia-Yi Tan, Jo-Ern Wong, Boon-Teong Teoh, Vunjia Tiong, Juraina Abd-Jamil, Siti-Sarah Nor’e, Chee-Sieng Khor, Jefree Johari, Che-Norainon Yaacob, Mulya-Mustika-Sari Zulkifli, AsmaAnati CheMatSeri, Nur-Hidayana Mahfodz, Noor Syahida Azizan, Sazaly AbuBakar

**Affiliations:** 1 Tropical Infectious Diseases Research and Education Centre (TIDREC), Higher Institution Centre of Excellence (HICoE), Universiti Malaya, 50603 Kuala Lumpur, Malaysia; 2 Department of Medical Microbiology, Faculty of Medicine, Universiti Malaya, 50603 Kuala Lumpur, Malaysia

**Keywords:** Epidemiology, SARS-CoV-2

## Abstract

The COVID-19 pandemic first emerged in Malaysia in Jan 2020. As of 12th Sept 2021, 1,979,698 COVID-19 cases that occurred over three major epidemic waves were confirmed. The virus contributing to the three epidemic waves has not been well-studied. We sequenced the genome of 22 SARS-CoV-2 strains detected in Malaysia during the second and the ongoing third wave of the COVID-19 epidemic. Detailed phylogenetic and genetic variation analyses of the SARS-CoV-2 isolate genomes were performed using these newly determined sequences and all other available sequences. Results from the analyses suggested multiple independent introductions of SARS-CoV-2 into Malaysia. A new B.1.524(G) lineage with S-D614G mutation was detected in Sabah, East Malaysia and Selangor, Peninsular Malaysia on 7th October 2020 and 14th October 2020, respectively. This new B.1.524(G) group was not the direct descendant of any of the previously detected lineages. The new B.1.524(G) carried a set of genetic variations, including A701V (position variant frequency = 0.0007) in Spike protein and a novel G114T mutation at the 5’UTR. The biological importance of the specific mutations remained unknown. The sequential appearance of the mutations, however, suggests that the spread of the new B.1.524(G) lineages likely begun in Sabah and then spread to Selangor. The findings presented here support the importance of SARS-CoV-2 full genome sequencing as a tool to establish an epidemiological link between cases or clusters of COVID-19 worldwide.

## Introduction

The World Health Organization declared a newly emerged novel severe pneumonia of unknown etiology that spread out from Hubei Province, China as a pandemic disease named COVID-19 on 11th March 2020^1^. To date, over 222 million COVID-19 cases with more than 4.5 million deaths were reported^2^. The etiologic agent causing the pandemic is a novel beta coronavirus, severe acute respiratory syndrome coronavirus-2 (SARS-CoV-2), which was initially named as the 2019 novel coronavirus (2019-nCoV)^3^. It is a single-stranded positive-sense RNA virus, with a genome of 29.9 kb in length^4^. Genetically, the SARS-CoV-2, together with severe acute respiratory syndrome coronavirus-1 (SARS-CoV-1) and the SARS-CoV-related bat's viruses, formed the *sarbecovirus* subgenus under the family *Coronaviridae*^5^. At the beginning of the pandemic, much effort was devoted towards determining the probable origin of the SARS-CoV-2^6–8^. The high genome sequence similarity between SARS-CoV-2 with coronaviruses recovered in bats and pangolins, suggests its potential natural zoonotic origin^6,8^. Given the massive global transmission of the virus and rapid increment of the total number of SARS-CoV-2 genome sequences in the public databases^9^, there has been growing interest and extensive studies exploring the genome information to facilitate the understanding of the spatiotemporal characteristics of the virus transmission within and across different continents and populations^10–12^. SARS-CoV-2 rapidly diverges into several lineages with genetic variations spanning the genome, supporting specific genomics sites evolving under positive selection^13^. Hence, continuous monitoring of the genetic variations is critical and crucial to detect and track the emergence of mutations that could have a potential impact on virus pathogenicity^14^, diagnostic test accuracy^15^, vaccine efficacy^16^ and the elucidation of SARS-CoV-2 evolution pattern.

The first four cases of COVID-19 in Malaysia were reported on 25th January 2020 from visiting Chinese nationals^17^. This marked the beginning of the first wave of the COVID-19 pandemic which lasted until the end of February 2020 with at least 10 days of zero cases recorded^18,19^. During the first wave, almost all the cases were associated with prior travel or contact with someone who has been to China. In early March 2020 an outbreak amongst at least 16,000 attendees of a religious gathering in Kuala Lumpur, fuelled a nationwide spread of the infection and this triggered the second wave of the pandemic with over 100 daily cases reported^20^. To contain the spread, the country implemented a nationwide Movement Control Order (MCO) on18th March 2020^20^. During the MCO, Malaysians were barred from traveling overseas, and inter-and intra-state travel was restricted to only those providing critical services. At the same time, the operation of all educational institutions, government agencies, and non-essential businesses temporarily ceased. These strict control strategies successfully contained the spread of SARS-CoV-2 and flattened the COVID-19 epidemic curve^21^ and Malaysia entered the recovery MCO (RMCO) phase on 10th Jun 2020^22^. The daily number of COVID-19 cases remained relatively low (< 20) and confined to limited locations^23^. Beginning from the end of July, however, COVID-19 cases steadily increased with a major upsurge of cases in different states recorded at the end of September 2020^24^. The extensive traveling of individuals canvassing for votes between Sabah and the different states in Malaysia during a local but statewide election was suggested as the major cause for the surge in COVID-19 cases. Sabah is a state in Malaysia situated on the Borneo island separated from Peninsular Malaysia by ~ 1600 km across the South China Sea. Beginning from 8th October 2021 Malaysia officially entered the third wave of the COVID-19 epidemic with reported daily cases of over 2000 and substantial community transmissions in Sabah, Selangor, and Kuala Lumpur. The MCO 2.0 was implemented on13th Jan 2021 to curb the spread of the infection^25^ and the National COVID-19 Immunization Program was launched on 24th February 2021^26^. The percentage of the adult population who has received two doses of immunization reached 50% by 8^th^ Sept, 2021. However, the number of cases continues to escalate reaching over 19,000 daily positive cases^27^. As of 12^th^ Sept 2021, there were 1,979,698 confirmed COVID-19 cases in Malaysia. Despite the high number of cases not much has been reported on the SARS-CoV-2 strains causing these outbreaks^21,28^.

To better understand the evolution of SARS-CoV-2 in Malaysia, we report here the full genome sequencing of the SARS-CoV-2 obtained in April and October 2020 during the second and third wave of the COVID-19 epidemic. Using the genome-scale analysis, we compared and investigated the genetic traits and phylogenetic relationships of these newly sequenced SARS-CoV-2 strains against other reported Malaysia strains. Specially, we sought to answer the local transmission and circulation pattern of the SARS-CoV-2. With limited available full genome sequences, the study's findings allowed us to resolve the relationship between isolates detected from the different states and times. It provides a better insight into the epidemiological link and elucidated the possible link of the actively circulating strains/clusters in the community. We highlight the importance and usefulness of the complete virus genome sequence analysis as a complementary tool with our current contact tracing approach for a more comprehensive and targeted disease monitoring and prevention intervention.

## Materials and methods

### Ethics statement

The study was approved and the need for informed consent was waived by the Medical Ethics Committee of the University Malaya Medical Centre (MREC ID no.: 20201228-9626). All methods were performed following all the relevant guidelines and regulations.

### Virus strains

The Tropical Infectious Diseases Research and Education Centre (TIDREC), Universiti Malaya (UM), is one of the research centers that participated in the Ministry of Science, Technology, and Innovation (MOSTI)-Ministry of Higher Education (MOHE) COVID-19 testing initiative. The SARS-CoV-2 samples used in the current study originated from samples tested positive by Real-time qRT-PCR during the diagnostic processes. RNA material received was anonymous without any identifier linked to the sample. The sample collection represented a random subset of the circulating SARS-CoV-2 strains during the period between April to May 2020 (second COVID-19 epidemic wave) and October 2020 (third COVID-19 epidemic wave).

### Sample preparation, genome sequencing, and assembly

All laboratory activities involving the COVID-19 testing were conducted following biosafety practices designed for COVID-19 testing in a biocontainment level II (BSL-2) laboratory. The amplicon libraries encompassing the SARS-CoV-2 full genome were prepared using the Ion AmpliSeq™ SARS‑CoV‑2 Research Panel (Ion Torrent, Thermo Scientific) with minor modification as previously described^29^. Briefly, the cDNA was generated using Superscript® IV Reverse Transcriptase (Invitrogen, Thermo Scientific). The generated cDNAs were subjected to SARS-CoV-2 full genome amplification using two primer pools of the Ion AmpliSeq™ SARS‑CoV‑2 Research Panel, according to the manufacturer's recommendation amplification protocol. The adaptors and barcodes were ligated to the pooled amplicon libraries using Ion AmpliSeq™ Library Kit Plus (Ion Torrent, Thermo Scientific). The barcode-ligated amplicon libraries were subjected to another round of amplification and clean-up processes prior to sequencing template preparation. The sequencing template was prepared using Ion PGM™ Hi-Q™ View OT2 Kit, and the full genome sequencing was performed using the Ion Personal Genome Machine (PGM) with 550 flows. The generated reads were mapped to SARS-CoV-2 reference strains, Wuhan-Hu-1 (GenBank accession number: MN908947). The level of sequence coverage on the target genome regions was performed using coverageAnalysis v5.12.0.0, as implemented in Torrent Suite™ Software 5.12. Genome assembly was conducted using the IRMAreport v1.2.1.0 (Ion Torrent, Thermo Scientific).

### Multiple sequence alignment, variant calling, and parsimony sites analysis of SARS-CoV-2

The SARS-CoV-2 sequences generated in this study, along with genome sequences of other Malaysian SARS-CoV-2 retrieved from the GISAID, were aligned to Wuhan-Hu-1 (MN908947.3). The informative variant sites were retrieved from the multiple sequence alignment (MSA) of this Malaysian SARS-CoV-2 using MEGA X^30^. The frequency of identified mutation variants globally was observed using mutation list listed in COVID CoV Genetics (COVID CG, https://covidcg.org/), China National Center for Bioinformation (CNCB)—2019 Novel Coronavirus Resource (2019nCoVR; https://bigd.big.ac.cn/ncov/)^31^, and CoV-GLUE (http://cov-glue.cvr.gla.ac.uk/)^32^.

### Clade/lineage assignment of Malaysian SARS-CoV-2

The clade/lineage assignment of the Malaysia SARS-CoV-2 was determined based on two major databases or SARS-CoV-2 nomenclatures previously proposed^9,33^. The SARS-CoV-2 clade of the newly sequenced Malaysia SARS-CoV-2 strains was first assigned based on the marker variants retrieved from GISAID on 6 November 2020^9^. The more detailed SARS-CoV-2 lineage was assigned by the Phylogenetic Assignment of Named Global Outbreak LINeages (PANGOLIN) tool (Version 2.1.7, 2021-01-20, https://pangolin.cog-uk.io/).

### Phylogenetic analysis of Malaysian SARS-CoV-2

The near-complete genome sequences (66–29,674) were extracted from the MSA of the Malaysia SARS-CoV-2. Sequences with low coverage and gaps were removed from the analysis. The resulting datasets consisting of 106 Malaysia SARS-CoV-2 strains were used for the phylogenetic tree reconstruction (Supplementary Table 1). The phylogeny of Malaysia SARS-CoV-2 was estimated using the Bayesian Markov Chain Monte Carlo (MCMC) approach, as implemented in BEAST 2.6^34^. The Generalised Time-Reversible model with the invariant site (GTR + I) was selected using the Akaike Information Criterion (AIC) as implemented in jModel Test 2.1.4^35^. The analysis was performed under a strict molecular clock model with an MCMC chain length of 20 million samplings every 2,000 generations. The resulting MCMC trace file was analyzed and visualized using Tracer Version 1.7^36^. The maximum clade credibility (MCC) tree was produced using TreeAnnotator 2.3.2^37^ and visualized using FigTree V1.4.4^38^.

## Results

### Genome structure, geographical distribution and clade assignment of SARS-CoV-2 strains

In the current study, 22 complete and near-complete genome sequences of SARS-CoV-2 strains detected in Malaysia were generated (Table 1). The genome sequences encompassing at least from positions 26 at the 5’-untranslated region (5’UTR) to 29,847 at the 3’-untranslated region (3’UTR) according to the nucleotide position of the Wuhan-Hu-1 genome sequence (MN908947.3). All SARS-CoV-2 complete genome sequences (16 out of 22) in this study possessed a similar genome structure with the reference sequence, Wuhan-Hu-1 with no insertion or deletion detected within the positions that ranged from nucleotide 26 to 29,847. Among these 22 genome sequences, 12 were detected between April to May 2020, representing circulating SARS-CoV-2 strains during the second wave of the COVID-19 epidemic in Malaysia. Most of the second wave’s SARS-CoV-2 strains were detected in Selangor, a state in Peninsular Malaysia. Only 1 sample, 4Apr20-3-Hu/2020, was detected from Negeri Sembilan, a neighboring state to Selangor (Table 1). While the other ten SARS-CoV-2 strains were detected from Sabah and Selangor, representing a subset of circulating strains detected during the initial phase of the third COVID-19 epidemic wave.

**Table 1:** Clade and lineage assignments of SARS-CoV-2 strains.

No	SARS-CoV-2 strains	Location	Epidemic wave	PANGOLIN Lineage	GISAID Clade	Genetic markers for GISAID clade assignment									
						241	3037	23,403	8782	11,083	22,227	25,563	26,144	28,144	28,882
	Reference genome					**C**	**C**	**A**	**C**	**G**	**C**	**G**	**G**	**T**	**G**
1	4Apr20-3-Hu/2020	Negeri Sembilan	2	B.1	G	T	T	G							
2	5Apr20-64-Hu/2020	Selangor	2	B.1.250	G	T	T	G							
3	21Apr20-101-Hu/2020	Selangor	2	B.6.1	O	T				T					
4	21Apr20-106-Hu/2020	Selangor	2	B.6.1	O	T				T					
5	21Apr20-128-Hu/2020	Selangor	2	B.6.1	O	T				T					
6	21Apr20-130-Hu/2020	Selangor	2	B.6.1	O	T				T					
7	21Apr20-209-Hu/2020	Selangor	2	B.6.1	O	T				T					
8	21Apr20-211-Hu/2020	Selangor	2	B.6.1	O	T				T					
9	21Apr20-224-Hu/2020	Selangor	2	B.6.1	O	T				T					
10	21Apr20-232-Hu/2020	Selangor	2	B.6.1	O	T				T					
11	21Apr20-236-Hu/2020	Selangor	2	B.6.1	O	T				T					
12	2May20-132-Hu/2020	Selangor	2	B.6.6	O					T					
13	7ct20-45-Hu/2020	Sabah	3	B.1.524	G	T	T	G							
14	7Oct20-83-Hu/2020	Sabah	3	B.1.524	G	T	T	G							
15	7OCT20-135-Hu/2020	Sabah	3	B.1.524	G	T	T	G							
16	7Oct20-152-Hu/2020	Sabah	3	B.1.524	G	T	T	G							
17	7Oct20-193-Hu/2020	Sabah	3	B.1.524	G	T	T	G							
18	14OCT20-136-Hu/2020	Selangor	3	B.1.524	G	T	T	G							
19	14OCT20-158-Hu/2020	Selangor	3	B.1.524	G	T	T	G							
20	14OCT20-183-Hu/2020	Selangor	3	B.1.524	G	T	T	G							
21	14OCT20-210-Hu/2020	Selangor	3	B.1.524	G	T	T	G							
22	14OCT20-219-Hu/2020	Selangor	3	B.1.524	G	T	T	G							

The clade/lineage of SARS-CoV-2 strains was assigned based on the GISAID clade and PANGOLIN Lineage assignments. Currently, there are seven GISAID assigned SARS-CoV-2 clades based on a list of ten genetic markers (Table 1 and Supplementary Table 2). Samples that did not cluster under these seven major GISAID clades will be denoted as others (O). Based on the GISAID clade assignment, the samples detected during early April 2020 (4Apr20-3-Hu/2020 and 5Apr20-64-Hu/2020), and all samples detected in October 2020 (third epidemic wave) denoted clade G (Table 1), possessed genetic variations at C241T, C3037T, and A23403G. Using the Pangolin COVID-19 Lineage Assigner, these clade G strains belonged to the same lineage, B.1.524 (Table 1). Samples obtained on 21 April 2020 and 2 May 2020 did not cluster under any of the seven major GISAID clades, hence, denoted as O. All samples obtained on 21 April 2020 carried two nucleotide changes C241T and G11803T, while sample obtained on 2 May 2020 (2May20-132-Hu/2020) only possessed G11803T. Based on the Pangolin assignment, all samples detected on 21 April 2020, denoted as lineage B.6.1 and sample detected on 2 May 2020 sample was assigned under B.6.6.

### Phylogenetic relationships and molecular signature of Malaysian SARS-CoV-2

The phylogenetic relationships of SARS-CoV-2, detected in Malaysia, were examined using a phylogenetic tree reconstructed using near-complete genome sequences ranging from nucleotide 66 to 29,674. The tree consisted of 106 SARS-CoV-2 strains (16 generated from this study and 90 retrieved from GISAID) detected between 28 January 2020 and 14 October 2020. For a better illustration of the circulation of different SARS-CoV-2 clades/lineages in Malaysia, we investigated and reported the relationships of SARS-CoV-2 in accordance with the three COVID-19 epidemic waves. The first COVID-19 epidemic wave in Malaysia started on 24 January 2020 and ended on 16 February^39^. The second COVID-19 epidemic wave started on 27 February^39^ with no clear indication of the end date before the third COVID-19 epidemic wave announcement on 8 October^40^. The Religious Gathering Cluster, the major contributor of the second COVID-19 epidemic wave, ended on 8 July^23^, and the cases remained relatively low before the emergence and spread linked to clusters in Kedah and Sabah. Therefore, to ease the explanation, we suggested a period between 27 February to 8 July as the second COVID-19 epidemic wave and 9 July to 7 October as the pre-emergence phase of the third epidemic wave of COVID-19 in Malaysia.

### First COVID-19 epidemic wave in Malaysia (25 January 2020–16 February 2020)

**Figure 1:**
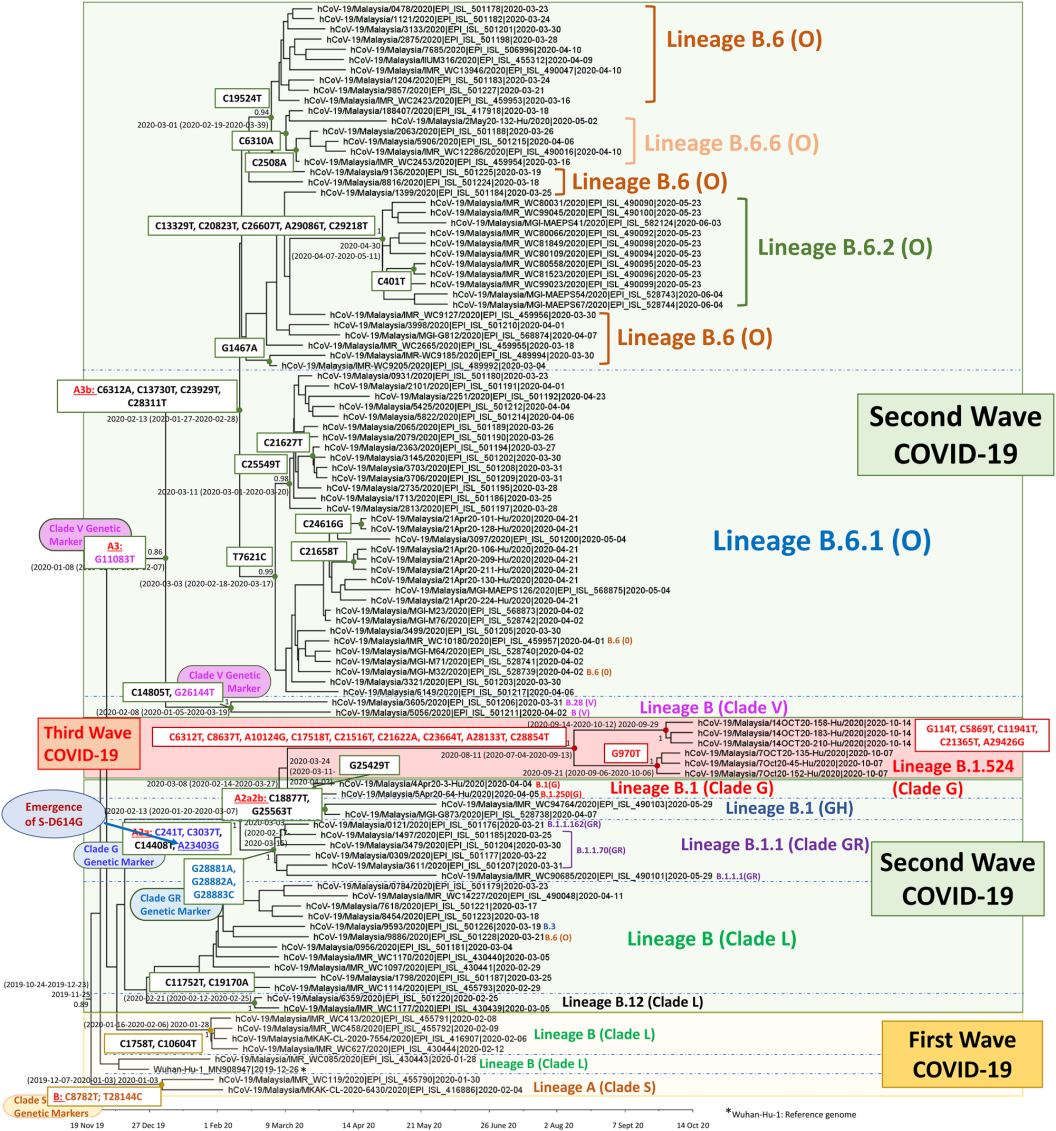
Maximum clade credibility (MCC) tree of SARS-CoV-2 isolated in Malaysia. The phylogenetic tree was constructed using the near-complete genome of SARS-CoV-2 strains (position 66-.29674). The major circulating lineages in Malaysia and their respective molecular signatures were shown.

Our phylogenetic analysis showed that SARS-CoV-2 strains of both SARS-CoV-2 lineages, lineage A and lineage B, were detected in COVID-19 patients during the first COVID-19 epidemic wave (Fig. 1). The lineage A (GISAID clade S) consisted of two strains, the hCoV-19/Malaysia/IMR WC119/2020 and the hCoV-19/Malaysia/MKAK-CL-2020-6430/2020, detected on 30 January 2020 and 4 February, respectively. Besides the GISAID clade S-specific genetic markers, C8782T and T28144C, there were no other shared mutations between these two samples. Despite lineage A still actively circulating in other parts of the world, no other lineage A strains were reported in Malaysia after the first wave. The lineage B (GISAID Clade L) was closely related to the first sequenced SARS-CoV-2 strain, Wuhan-Hu-1 (Fig. 1). Malaysia’s lineage B(L) strains detected between 6 to 12 February clustered closely to form a distinct subgroup. These strains shared two missense mutations, the C1758T which encoded for amino acid substitution of alanine to valine at position 318 of nsp2, and the C10604T encoded for amino acid substitution proline with serine at position 184 of nsp5 (Table 2). These mutations, however, were not observed in other B(L) strains detected after the first wave, consistent with epidemiological data that early introduction of SARS-CoV-2 during the first wave was successfully contained. We looked into the time of emergence of these mutations (https://bigd.big.ac.cn/ncov/); the first strain which carried the C1064T (nsp5-P184S) was reported in hCoV-19/Beijing/BJ53/2020 detected on 24 January 2020 from Beijing, while the first strain that carried both C1758T/C10064T was hCoV-19/Malaysia/MKAK-CL-2020–7554/2020 detected in Malaysia on 6 February 2020.

**Table 2:** List of specific mutations of Malaysia SARS-CoV-2 strains.

Genetic variation	Lineage/Clade	Effect	Amino acid substitution			Analysis of mutation sites (Data Retrieved from CNBG on 9 Nov 2020)		
			Reference	Mutant	Mutant	Number of isolates with mutations (Any type of mutations)	Number of isolates with the specific mutation	Position variant frequency
C8782T	A(S)	Synonymous	S	–	nsp4-76S	4209	4401	0.0375
T28144C		Missense	L	S	ORF8-L84S	4204	4402	0.0374
C1758T	B(L)	Missense	A	V	nsp2-A318V	401	396	0.0036
C10604T		Missense	P	S	nsp5-P184S	35	34	0.0003
C11752T	B.12(L)	Synonymous	L	–	nsp6-260L	88	101	0.0008
C19170A		Missense	F	L	nsp14-F377L	637	51	0.0057
C241T	B.1(G) B.1.1(GR) B.1.1.1(GR) B.1(GH) B.1.160(GH)	–	–	–	–	100,656	103,714	0.8965
C3037T		Synonymous	F	–	nsp3-106F	101,561	104,625	0.9045
C14408T		Missense	P	L	nsp12-P323L	101,424	104,458	0.9033
A23403G		Missense	D	G	S-D614G	101,615	104,674	0.905
G28881A	B.1(GH) B.1.160(GH)	Missense	R	K	N-R203K	42,724	43,788	0.3805
G28882A						42,543	43,603	0.3789
G28883C		Missense	G	R	N-G204R	42,530	43,599	0.3789
C18877T	B.1.1(GR) B.1.1.1(GR)	Synonymous	L	–	nsp14-280L	6140	6210	0.0547
G25563T		Missense	Q	H	ORF3a-Q37H	26,880	27,738	0.2394
G25429T	B.1(G) *Apr 2020	Missense	V	L	ORF3a-V13L	994	994	0.0089
C6312T	B.1.524(G) *Oct 2020	Missense	T	I	nsp3-T1198I	1127	80	0.01
C8637T		Missense	T	I	nsp4-T28I	13	7	0.0001
A10124G		Missense	T	A	nsp5-T24A	6	6	0.0001
C17518T		Missense	L	F	nsp13-L428F	106	106	0.0009
C21516T		Synonymous	N	–	nsp16-858 N	101	100	0.0009
C21622A		Synonymous	T	–	S-20 T	114	10	0.001
C23664T		Missense	A	V	S-A701V	74	77	0.0007
A28133T		Synonymous	T	-	ORF8-80 T	173	183	0.0015
C28854T		Missense	S	L	N-S194L	5558	5585	0.0495
G970T	B.1.624(G) *Oct 2020 (Sabah)	Missense	E	D	nsp2-E55D	40	1	0.0004
G114T	B.1.524(G) *Oct 2020 (Selangor)	–	–	–	–	1	0	0
C5869t		Synonymous	Y	–	nsp3-1050Y	115	113	0.0001
C11941T		Synonymous	V	–	nsp7-33 V	73	66	0.0007
C21365T		Missense	P	L	nsp16-P236L	33	30	0.0003
A29426G		Missense	R	G	N-R385G	2	1	0
G11083T	B.2(V) B.6 (O)	Missense	L	F	nsp6-L37F	7972	7938	0.071
C14805T	B.2(V)	Synonymous	Y	–	nsp12-455Y	3295	3389	0.0293
G26144T		Missense	G	V	ORF3a-G251V	2467	2520	0.022
C6312A	B.6(O)	Missense	T	K	nsp3-T1198K	1127	1052	0.01
C13730T		Missense	A	V	nsp12-A97V	1200	1202	0.0107
C23929T		Synonymous	Y	–	S-789Y	1022	1052	0.0091
C28311T		Missense	P	L	N-P38L	1275	1319	0.0114
T7621C	B.6(O)	Synonymous	C	–	nsp3-1634C	29	0	0.0003
C21658T		Synonymous	F	–	S-32F	91	70	0.0008
C24616G		Missense	I	M	S-I1018M	23	0	0.0002
C25549T		Missense	L	F	ORF3a-L53F	322	318	0.0029
C21627T		Missense	T	I	S-T22I	74	63	0.0007
G1467A	B.6(O)	Missense	G	D	nsp2-G221D	7	7	0.0001
C13329T	B.6(O)	Missense	T	I	nsp10-T102I	113	113	0.001
C20823T		Synonymous	N	–	nsp16-55 N	–	–	–
C26607T		Missense	L	F	M-L29F	–	–	–
A29086T		Synonymous	T	–	N-271 T	–	–	–
C29218T		Synonymous	F	–	N-215F	86	75	0.0008
C401T		Missense	L	F	nsp1-L46F	21	15	0.0002
C19524T	B.6(O)	Synonymous	L	–	nsp14-495L	1035	1073	0.0092
C6310A		Missense	S	R	nsp3-S1197R	722	535	0.0064
C2508A		Missense	P	H	nsp2-P568H	138	6	0.0012

### Second COVID-19 epidemic wave in Malaysia (27 February–8 July 2020)

The majority of the samples (93 out of 106) used in this study were obtained during the second COVID-19 epidemic wave (Fig. 1). All the samples clustered under lineage B. Our phylogenetic analyses showed that the samples were segregated into two major groups before they were delineated into multiple sub-lineages. The first group clustered closely with B(L) strains detected during the first COVID-19 epidemic phase (Fig. 1), representing strains assigned as lineages B(L), B.1(G, GH, GR), and B.12(L). The second group represented previously assigned strains under lineage B(V) and B.6(O).

### B(L)/B.1(G,GH,GR)-associated subgroups

The B/B.1/B.12 group consisted of strains detected between 25 February to 14 October 2020 (Fig. 1). On the phylogenetic tree, the strains segregated into two major subgroups consisting of strains assigned under B/B.12 and B.1. The B/B.12 subgroup was further delineated into groups which corresponded to lineage B.12 and lineage B. These B/B.12 strains, however, were all assigned as clade L under GISAID except hCoV-19/Malaysia/9886/2020(B.6(O)). Malaysia’s lineage B.12 consisted of two strains, the hCoV-19/Malaysia/6359/2020 and hCoV-19/Malaysia/IMR WC1177/2020. These B.12(L) strains contained two genetic variations, C11752T and C19170A (Fig. 1 and Table 2). These two mutations were first reported in hCoV-19/Japan/PG-0015/2020 (EPI_ISL_479799), a strain detected from Hokkaido, Japan, on 20 January 2020. This was consistent with our analysis that the Most Recent Common Ancestor (MRCA) of these Malaysia’s B.12 strains could have dated back to 21 January 2020 (95% HPD: 12 February–25 February 2020). These two linked mutations were common genetic traits for strains detected in Hokkaido, Japan, from January to March 2020, suggesting the possible Japan-origin of Malaysia’s B.12 strains. Unlike the B.12 subgroup, the other second wave’s strains clustered under lineage B did not have any common genetic variation, indicating they could have been imported independently from different sources.

The second subgroup was a group of lineage B.1 strains and their descendants, comprising the strains detected between 21 March to 14 October 2020 (second and third COVID-19 epidemic waves, Fig. 1). All strains clustered under this group, including those sequenced in the current study, possessed the three GISAID clade G genetic variants, C241T, C3037T, and A23403G with an additional mutation C14408T. The C14408T is a common mutation used to define B.1 in the Pangolin system, and it is also used to define a haplogroup A2a4 (another SARS-CoV-2 clustering system)^10^. The C14408T and A23403G were missense mutations (Table 2). The C14408T was encoded for substitution of proline with leucine at position 323 in nsp12 of ORF1ab (nsp12-P323L). The A23403G was encoded for substitution of aspartic acid with glycine in the S-614 (S-D614G). So far, only strains that fell within this B.1-associated lineage carried this S-D614G amino acid substitution. The strains that possessed all four mutations (C241T-C3037T-C14408T-A23403T) were actively circulating, especially in Europe (https://bigd.big.ac.cn/ncov/) before its first documented detection in Malaysia in late March, 2020 (Fig. 1). Our data showed that this B.1-associated lineage further segregated into four subgroups, representing strains of B.1.1.X(GR), B.1(GH), and B.1(G), and B.1.524(G) groups. Strains that possessed additional three genetic markers G28881A, G28882A, and G28883C, clustered under clade GH, strains which possessed G25563T was assigned as clade GR, and the strains that presented without these additional genetic variations remained as Clade G. Malaysia’s B1.1.X(GR) group comprised six strains detected between 21 March to 29 May 2020. There was no additional mutations shared among this group besides the clade-specific mutations. Malaysia’s GH group comprised two strains, hCoV-19/Malaysia/MGI-G873/2020, detected on 7 April 2020, and hCoV-19/Malaysia/IMR WC94764/2020, detected on 29 May 2020. Both strains shared an additional genetic variation, C18877T, a synonymous mutation. The assigned B.1(G) strains segregated into two distinct subgroups. The first subgroup consisted of two strains sequenced in this study, hCoV-19/Malaysia/4Apr20-3-Hu/2020 detected from Negeri Sembilan on 4 April 2020 and hCoV-19/Malaysia/5Apr20-64-Hu/2020 detected in Selangor on 5 April 2020. An additional shared genetic variation, G25429T encoded for an amino acid substitution of valine to leucine at position 13 of the ORF3a (ORF3a-V13L), was observed. These two spatially separated ORF3a-L13-bearing B.1(G) strains could have descended from a common ancestor carrying the 25429 T mutation. The mutation at this position is relatively rare, with a variation frequency of less than 0.01 (Table 2).

### B.2(V)/B.6(O) subgroups

The second major group was B/B.6 lineage (Fig. 1). All strains within this B.2/B.6 lineage shared genetic variation G11083T, encoded for amino acid changes from leucine to phenylalanine at position 37 of nsp6 in ORF1ab. It was a common mutation detected in strains circulating in China in January 2020; the earliest isolates with this mutation dated back to 17 January 2020 (https://bigd.big.ac.cn/ncov/). These T11803-bearing strains segregated into two groups corresponded to lineage B(clade V) and lineage B.6 (No specific GISAID clade was assigned, denoted as O, referring to others).

Two B(V) strains were detected in Malaysia on 31 March and 2 April 2020 (Fig. 1). In addition to the T11803, both strains possessed additional two nucleotide variations, C14805T and G26144T. The G11083T and G26144T were genetic markers for the assignment of GISAID Clade V. While the C14805T was an additional genetic trait present in this group. The C14805T was a synonymous mutation that was not originally present in China during the early spread of the virus, suggesting this mutation could have accumulated in the SARS-CoV-2 gene pool outside of China. These three linked mutations, however, were detected in strains detected in England and Korea beginning at the end of Jan and early Feb 2020, indicating the widespread distribution of the ancestor strains (https://covidcg.org/). There was no B(V) strain detected in Malaysia after 2 April 2020.

The B.6 (O)-associated lineage formed the largest group of Malaysia’s SARS-CoV-2 phylogenetic tree, comprised of strains detected from 4 March to 4 June 2020. The SARS-CoV-2 strains sequenced in this study (21 April–2 May 2020) clustered within this subgroup. All B.6 strains within this group contained four additional genetic variations; C6312A, C13730T, C23929T, and C28311T in addition to G11083T mutation. These five mutations were probably linked mutations as these mutations were detected simultaneously in strains recovered from different countries in March 2020 (https://bigd.big.ac.cn/ncov/). These A6312A-T11083-T13730-T23929-T28311-bearing strains were segregated into multiple distinct subgroups with the presence of several unique genetic traits. Sequential accumulation of these mutations could have reflected the transmission path of SARS-CoV-2 in the local community. For example, the B.6.1(O) subgroup was also characterized by a mutation, T7621C. This T7621C mutation was a synonymous mutation detected in 29 strains, mainly from Malaysia and Brunei (https://bigd.big.ac.cn/ncov/variation/annotation/variant/7612), suggesting that this is a unique mutation that occurred in this region and could have originated from a single origin. These C7621-bearing strains were further delineated into two groups differentiated by an additional mutation, C25549T. Our samples obtained in mid-Apr 2020 clustered within the C7621-C2554T-groups, with additional mutations detected in some of the samples. Most of these samples were detected from individuals with travel histories to Indonesia and India. There was, however, no distinct spatial clustering of the samples. Samples’ collection time for the C25549-bearing and T25549-bearing groups, however, overlapped, indicating at least two independent and simultaneous transmission chains. Within B.6(O), another group with the additional mutation, C19524T, was observed; subsequently, an additional C6210A and then C2508A was detected in a subset of this group (B.6.6(O)). The B.6.6(O) comprised of samples linked to the identified Seri Petaling Gathering Cluster^41^. The T19524 and T19524T-A6210 strains were also detected in neighboring countries including Singapore, Thailand, and Australia (https://bigd.big.ac.cn/ncov/) but not the T19524-A6210-A2508 strains, suggesting the C2508A could be a mutation that accumulated in the SARS-CoV-2 gene pool during the second COVID-19 epidemic wave in Malaysia.

### Third COVID-19 epidemic wave in Malaysia (from 8 October)

Our phylogenetic results showed that all samples detected in Oct 2020 (Sabah and Selangor strains) clustered together within lineage B.1.524(G). Although these third epidemic wave strains clustered closely with strains detected in early April (hCoV-19/Malaysia/4Apr20-3-Hu/2020 and hCoV-19/Malaysia/5Apr20-64-Hu/2020), the genetic analysis showed that the Oct 2020 strains were not a direct descendant of April’s B.1(G) group. This is because the genetic trait (T25429) was not observed in the genome of the October 2020’s strains. Sequence analysis showed a common ancestor of B.1.524 lineage (T241-T3037-T14408-G23403), with the acquisition of nine mutations (C6312T, C8637T, A10124G, C17518T, C21516T, C21622A, C23664T, A28133T, and C28854T) that could have seeded transmission clusters of the two closely related groups in Sabah and Selangor (Tables 2 and 3). The newly emerged B.1.524(G) strains were likely the descendants of the same ancestor with the MRCA and could have dated back to 11 August 2020 (95% HPD: 4 July–15 September 2020). All nine third epidemic wave genetic variations reported herein were reported in the open reading frame (ORF) region of the genome. Among the nine variations, six (C6312T, C8637T, A10124G, C17518T, C23664T, and C28854T) caused amino acid substitutions (nsp3-T1198I, nsp4-T28I, nsp5-T24A, nsp13-L428F, S-A701V, and N-S194L). The 288,854 within nucleocapsid gene was a more common mutation site, with a frequency of nearly 0.05, compared to the other eight sites (< 0.01; Table 2).

**Table 3:** List of mutations in SARS-CoV-2 strains detected in Malaysia in October 2020.

Isolates	Nucleotide position																			
	114	241	970	3037	5869	6312	8637	10,124	11,941	14,408	17,518	21,365	21,516	21,622	23,403	23,664	28,133	28,854	29,426	29,751
Wuhan-Hu-1 MN908947	G	C	G	C	C	C	C	A	C	C	C	C	C	C	A	C	A	C	A	G
MY.14OCT20-136-Hu/2020	T	T		T	T	T	T	G	T	T	T	T	T	A	G	T	T	T	G	T
MY.14OCT20-158-Hu/2020	T	T		T	T	T	T	G	T	T	T	T	T	A	G	T	T	T	G	T
MY.14OCT20-183-Hu/2020	T	T		T	T	T	T	G	T	T	T	T	T	A	G	T	T	T	G	T
MY.14OCT20-210-Hu/2020	T	T		T	T	T	T	G	T	T	T	T	T	A	G	T	T	T	G	T
MY.14OCT20-219-Hu/2020		T		T	T	T	T	G		T	T	T	T	A	G	T	T	T		T
MY.7Oct20-45-Hu/2020		T	T	T		T	T	G		T	T		T	A	G	T	T	T		
MY.7Oct20-83-Hu/2020		T		T		T	T	G		T	T		T	A	G	T	T	T		
MY.7OCT20-135-Hu/2020		T	T	T		T	T	G		T	T		T	A	G	T	T	T		
MY.7Oct20-152-Hu/2020		T	T	T		T	T	G		T	T		T	A	G	T	T	T		
MY.7Oct20-193-Hu/2020		T	T	T		T	T	G		T	T		T	A	G	T	T	T		

To understand the relationships between the Sabah and Selangor subgroups, we retrieved all the genetic variations detected in Sabah and Selangor strains (Table 3). The G970T variation that caused an amino acid substitution at E55D in nsp2 (nsp2-E55D) was a unique genetic variation present in all Sabah strains except for hCoV-19/Malaysia/7Oct20-83-Hu/2020. This suggested that the 7Oct20-83-Hu/2020 (G970-bearing strain) could have represented the ancestral group before the acquisition of the T970 mutation. The resulting amino acid substitution, nsp2-E55D was a rare mutation currently only detected in one strain globally (https://bigd.big.ac.cn/ncov/variation/annotation/variant/970). On the other hand, all Selangor strains detected in Oct 2020 except for 14OCT20-219-Hu/2020 possessed five additional genetic variations (Table 3). Based on our epidemiological data, all the Selangor strains detected in Oct except MY.14OCT20-219-Hu/2020 were likely linked. The MY.14OCT20-219-Hu/2020 lacked G114T, C11941T, and A29426G but possessed different mutations (C4423T and C16376T; Table 3), likely pointing to the presence of another transmission chain. Out of the five genetic variations, one was identified in the 5’UTR regions (G114T), three located within ORF1ab (C5869T, C11941T, and C21365T), and one in the N gene (A29426G). The C21365T caused an amino acid substitution of Proline for Leucine at position 236 in nsp16 of the ORF1ab (nsp16-P236L), while A29426G caused the substitution of arginine for glycine at position 385 in N (N-R385G). All five mutations occurred at the sites with low variation frequency (< 0.001). The G114T mutation at 5UTR detected in Selangor strains, was a novel mutation reported for the first time in our study.

## Discussion

Overall, our findings suggest that there was no sustained transmission of a single SARS-CoV-2 lineage in Malaysia. The initial introductions of SARS-CoV-2 during the first epidemic wave and early of the second wave could have been represented as Stage 1 COVID-19 transmission^42^, where only imported cases, with no localized community transmission, were recorded^18^. It was evidenced by the absence of similar virus strain or descendant lineages detected during analysis. The findings ascribed the targeted border control measures, rapid contact tracing, and isolation during the early phase of the COVID-19 epidemic was effective in containing the SARS-CoV-2 spread during the period between late Jan to Feb 2020. Substantial local transmission of COVID-19 in Malaysia, which started in March 2020 as a result of a subsequent new introduction. Our finding suggests that contrary to the earlier suggestion^43^, the S-G614 strains were already present in Malaysia as early as March 2020 instead of July 2020. Despite the S-G614 could have better epidemic potential, as illustrated in previous studies^37,38^, the spread of the S-G614 groups before July 2020 went unnoticed. The lineage B.6(O)-associated groups (S-D614 strains) were the most successful clade documented in Malaysia in year 2020 with high genetic diversity during the second wave of COVID-19 epidemic. The early segregation of distinct B.6(O) sub-lineages on the phylogenetic tree with unique subgroup-specific genetic mutations suggests multiple independent importations and virus dissemination events that contributed to the high genetic diversity of the B.6(O). The third wave isolates detected from Sept 2020 onwards, showed that the isolates detected in Sabah and Selangor, the two highly affected states were not the direct descendant of any of the previous clusters, including the major contributor of the second wave, lineage B.6(O)^21^. It is a new lineage, B.1.524(G), with unique genetic mutations to differentiate them from the previous circulating strains. With the data presented herein, we cannot conclude if the B.6(O) lineage, the major contributor of the second wave, has completely ceased. The presence data, however, provide evidence of the emerging of a new B.1.524(G) in Malaysia, implying this new B.1.524(G) lineage could be one of the contributors to the early phase of the on-going third wave COVID-19 epidemic.

The extensive human-to-human transmission of SARS-CoV-2 allows for the accumulation of mutations in the actively circulating virus pool^36^. Due to its polymerase proofreading ability^44^, SARS-CoV-2 has a relatively slower evolutionary rate than other RNA viruses^45^. Hence, the slower evolutionary rate of SARS-CoV-2 showed that as few as one mutation could provide enough information to discern the transmission dynamics, especially in local settings with limited individual movement. Detection of B.1.514(G) asynchronously in Sabah and Selangor, suggesting the surges of COVID-19 cases in Oct 2020 could have originated from a common ancestral strain. It is very likely the new B.1.524(G) strains represent a group of unsampled strains that could have circulated long enough to allow the accumulation of nine “stable” genetic variations. Epidemiological findings suggest that the surge in local transmission in Sabah starts in mid-Sept 2020^46^ preceded the increase of COVID-19 cases in Selangor. This is suggesting the spread of B.1.524(G) could have begun in Sabah. With the epidemiological data, the B.1.524(G) could have originated from Indonesia or the Philippines, involving transborder movement of undocumented immigrants into Sabah, Malaysia. Epidemiological investigation suggests that many reported clusters in Peninsular Malaysia, including in Selangor, originated from Sabah as a result of the massive domestic travel prior to the implementation of the travel restrictions. So far, the B.1.524(G) strains have been detected in Singapore and Australia. We do not know if these strains were imported from Malaysia or a similar source where the Malaysia strains originate.

## Conclusion

The phylogenetic and genetic variation study of the SARS-CoV-2 detected in Malaysia showed the emergence of B.1.524(G) group in Oct 2020. This B.1.524(G) served as one of the prevalent circulating lineages for the on-going localized transmission during the third COVID-19 epidemic. This B.1.524(G) is a new group that did not resemble any of the S-G614 strains previously introduced into Malaysia. Unique genetic variations observed in this new B.1.524(G) suggests it originated from a group of actively circulating sub-lineages that probably remained unsampled. Sequencing of more isolates from different clusters would reveal if this B.1.524(G) is the major contributor to the third wave COVID-19 epidemic. It will also allow for a better understanding of the evolutionary pattern of SARS-CoV-2 in Malaysia. The findings presented were also highlight the potential use of sequencing data as a complementary tool to establish an epidemiological link between cases or clusters.

## Supplementary Information

Supplementary Information.

## Abbreviations

COVID-19: Coronavirus disease 2019
ISPs: Ion SphereParticles
MOHE: Ministry of Higher Education
MOSTI: Ministry of Science, Technology, and Innovation
NGS: Next-generation sequencing
PGM: Personal genome machine
rRT-PCR: Real-time reverse-transcription polymerase chain reaction
SARS-CoV-2: Severe acute respiratory syndrome coronavirus 2
TIDREC: Tropical Infectious Diseases Research and Education Centre
UM: Universiti Malaya
