# Supplementary material for: Emergence of B.1.524(G) SARS-CoV-2 in Malaysia during the third COVID-19 epidemic wave
Source: Sci Rep. 2021 Nov 11;11:22105. doi: 10.1038/s41598-021-01223-4 (PMC8586159; doi:10.1038/s41598-021-01223-4)
Supplement: Supplementary file 1 — Supplementary Information. [file 41598_2021_1223_MOESM1_ESM.pdf]

Emergence of B.1.524(G) SARS-CoV-2 in Malaysia during the Third COVID-19 Epidemic Wave

Kim-Kee Tan[1], Jia-Yi, Tan[1], Jo-Ern Wong[1], Boon-Teong Teoh[1], Vunjia Tiong[1], Juraina Abd-Jamil[1], Siti-Sarah Nor'e[1], Chee-Sieng Khor[1], Jefree Johari[1], Che-Norainon Yaacob[1], Mulya-Mustika-Sari Zulkifli[1], AsmaAnati CheMatSeri[1], Nur-Hidayana Mahfodz[1], Noor Syahida Azizan[1], Sazaly AbuBakar[1,2*]

Supplementary Table 1: List of the Malaysia SARS-CoV-2 used in the analysis

SARS-CoV-2 strains	Lineage reassigned on 5 Nov 2020	COVID-19 epidemic wave in Malaysia
hCoV-19/Malaysia/MKAK-CL-2020-6430/2020|EPI ISL 416886|2020-02-04	A.1(S)	First wave
hCoV-19/Malaysia/IMR WC119/2020|EPI ISL 455790|2020-01-30	A (S)	First wave
hCoV-19/Malaysia/IMR WC085/2020|EPI ISL 430443|2020-01-28	B (L)	First wave
hCoV-19/Malaysia/IMR WC627/2020|EPI ISL 430444|2020-02-12	B (L)	First wave
hCoV-19/Malaysia/MKAK-CL-2020-7554/2020|EPI ISL 416907|2020-02-06	B (L)	First wave
hCoV-19/Malaysia/IMR WC458/2020|EPI ISL 455792|2020-02-09	B (L)	First wave
hCoV-19/Malaysia/IMR WC413/2020|EPI ISL 455791|2020-02-08	B (L)	First wave
hCoV-19/Malaysia/IMR WC1177/2020|EPI ISL 430439|2020-03-05	B.12(L)	Second wave
hCoV-19/Malaysia/6359/2020|EPI ISL 501220|2020-02-25	B.12(L)	Second wave
hCoV-19/Malaysia/IMR WC1114/2020|EPI ISL 455793|2020-02-29	B(L)	Second wave
hCoV-19/Malaysia/1798/2020|EPI ISL 501187|2020-03-25	B (L)	Second wave
hCoV-19/Malaysia/IMR WC1097/2020|EPI ISL 430441|2020-02-29	B (L)	Second wave
hCoV-19/Malaysia/IMR WC1170/2020|EPI ISL 430440|2020-03-05	B (L)	Second wave
hCoV-19/Malaysia/0956/2020|EPI ISL 501181|2020-03-04	B (L)	Second wave
hCoV-19/Malaysia/9886/2020|EPI ISL 501228|2020-03-21	B.6 (O)	Second wave
hCoV-19/Malaysia/9593/2020|EPI ISL 501226|2020-03-19	B.3 (L)	Second wave
hCoV-19/Malaysia/8454/2020|EPI ISL 501223|2020-03-18	B (L)	Second wave
hCoV-19/Malaysia/7618/2020|EPI ISL 501221|2020-03-17	B (L)	Second wave
hCoV-19/Malaysia/IMR WC14227/2020|EPI ISL 490048|2020-04-11	B (L)	Second wave
hCoV-19/Malaysia/0784/2020|EPI ISL 501179|2020-03-23	B (L)	Second wave
hCoV-19/Malaysia/IMR WC90685/2020|EPI ISL 490101|2020-05-29	B.1.1.1(GR)	Second wave
hCoV-19/Malaysia/3611/2020|EPI ISL 501207|2020-03-31	B.1.1 (GR)	Second wave
hCoV-19/Malaysia/0309/2020|EPI ISL 501177|2020-03-22	B.1.1 (GR)	Second wave
hCoV-19/Malaysia/3479/2020|EPI ISL 501204|2020-03-30	B.1.1 (GR)	Second wave
hCoV-19/Malaysia/1497/2020|EPI ISL 501185|2020-03-25	B.1.1 (GR)	Second wave
hCoV-19/Malaysia/0121/2020|EPI ISL 501176|2020-03-21	B.1.1.162 (GR)	Second wave
hCoV-19/Malaysia/MGI-G873/2020|EPI ISL 528738|2020-04-07	B.1 (GH)	Second wave

hCoV-19/Malaysia/IMR WC94764/2020|EPI ISL 490103|2020-05-29	B.1 (GH)	Second wave
hCoV-19/Malaysia/5Apr20-64-Hu/2020|2020-04-04	B.1.250 (G)	Second wave
hCoV-19/Malaysia/4Apr20-3-Hu/2020|2020-04-04	B.1 (G)	Second wave
hCoV-19/Malaysia/7Oct20-45-Hu/2020|2020-10-07	B.1.524 (G)	Third wave
hCoV-19/Malaysia/7Oct20-152-Hu/2020|2020-10-07	B.1.524 (G)	Third wave
hCoV-19/Malaysia/7OCT20-135-Hu/2020|2020-10-07	B.1.524 (G)	Third wave
hCoV-19/Malaysia/14OCT20-210-Hu/2020|2020-10-14	B.1.524 (G)	Third wave
hCoV-19/Malaysia/14OCT20-183-Hu/2020|2020-10-14	B.1.524 (G)	Third wave
hCoV-19/Malaysia/14OCT20-158-Hu/2020|2020-10-14	B.1.524 (G)	Third wave
hCoV-19/Malaysia/5056/2020|EPI ISL 501211|2020-04-02	B (V)	Second wave
hCoV-19/Malaysia/3605/2020|EPI ISL 501206|2020-03-31	B.28 (V)	Second wave
hCoV-19/Malaysia/6149/2020|EPI ISL 501217|2020-04-06	B.6.1 (O)	Second wave
hCoV-19/Malaysia/3321/2020|EPI ISL 501203|2020-03-30	B.6.1 (O)	Second wave
hCoV-19/Malaysia/MGI-M32/2020|EPI ISL 528739|2020-04-02	B.6 (O)	Second wave
hCoV-19/Malaysia/MGI-M71/2020|EPI ISL 528741|2020-04-02	B.6.1 (O)	Second wave
hCoV-19/Malaysia/MGI-M64/2020|EPI ISL 528740|2020-04-02	B.6.1 (O)	Second wave
hCoV-19/Malaysia/IMR WC10180/2020|EPI ISL 459957|2020-04-01	B.6 (O)	Second wave
hCoV-19/Malaysia/3499/2020|EPI ISL 501205|2020-03-30	B.6.1 (O)	Second wave
hCoV-19/Malaysia/MGI-M76/2020|EPI ISL 528742|2020-04-02	B.6 .1(O)	Second wave
hCoV-19/Malaysia/MGI-M23/2020|EPI ISL 568873|2020-04-02	B.6.1 (O)	Second wave
hCoV-19/Malaysia/21Apr20-224-Hu/2020|2020-04-21	B.6.1 (O)	Second wave
hCoV-19/Malaysia/MGI-MAEPS126/2020|EPI ISL 568875|2020-05-04	B.6.1 (O)	Second wave
hCoV-19/Malaysia/21Apr20-130-Hu/2020|2020-04-21	B.6.1 (O)	Second wave
hCoV-19/Malaysia/21Apr20-209-Hu/2020|2020-04-21	B.6.1 (O)	Second wave
hCoV-19/Malaysia/21Apr20-211-Hu/2020|2020-04-21	B.6.1 (O)	Second wave
hCoV-19/Malaysia/21Apr20-106-Hu/2020|2020-04-21	B.6.1 (O)	Second wave
hCoV-19/Malaysia/3097/2020|EPI ISL 501200|2020-05-04	B.6.1 (O)	Second wave
hCoV-19/Malaysia/21Apr20-128-Hu/2020|2020-04-21	B.6.1 (O)	Second wave
hCoV-19/Malaysia/21Apr20-101-Hu/2020|2020-04-21	B.6.1 (O)	Second wave
hCoV-19/Malaysia/2813/2020|EPI ISL 501197|2020-03-28	B.6.1 (O)	Second wave
hCoV-19/Malaysia/1713/2020|EPI ISL 501186|2020-03-25	B.6.1 (O)	Second wave
hCoV-19/Malaysia/2735/2020|EPI ISL 501195|2020-03-28	B.6.1 (O)	Second wave
hCoV-19/Malaysia/3706/2020|EPI ISL 501209|2020-03-31	B.6.1 (O)	Second wave
hCoV-19/Malaysia/3703/2020|EPI ISL 501208|2020-03-31	B.6.1 (O)	Second wave
hCoV-19/Malaysia/3145/2020|EPI ISL 501202|2020-03-30	B.6.1 (O)	Second wave
hCoV-19/Malaysia/2363/2020|EPI ISL 501194|2020-03-27	B.6.1 (O)	Second wave
hCoV-19/Malaysia/2079/2020|EPI ISL 501190|2020-03-26	B.6.1 (O)	Second wave
hCoV-19/Malaysia/2065/2020|EPI ISL 501189|2020-03-26	B.6.1 (O)	Second wave
hCoV-19/Malaysia/5822/2020|EPI ISL 501214|2020-04-06	B.6.1 (O)	Second wave
hCoV-19/Malaysia/5425/2020|EPI ISL 501212|2020-04-04	B.6.1 (O)	Second wave
hCoV-19/Malaysia/2251/2020|EPI ISL 501192|2020-04-23	B.6.1 (O)	Second wave
hCoV-19/Malaysia/2101/2020|EPI ISL 501191|2020-04-01	B.6.1 (O)	Second wave
hCoV-19/Malaysia/0931/2020|EPI ISL 501180|2020-03-23	B.6.1 (O)	Second wave

hCoV-19/Malaysia/IMR-WC9205/2020|EPI ISL 489992|2020-03-04	B.6 (O)	Second wave
hCoV-19/Malaysia/IMR-WC9185/2020|EPI ISL 489994|2020-03-30	B.6 (O)	Second wave
hCoV-19/Malaysia/IMR WC2665/2020|EPI ISL 459955|2020-03-18	B.6 (O)	Second wave
hCoV-19/Malaysia/MGI-G812/2020|EPI ISL 568874|2020-04-07	B.6 (O)	Second wave
hCoV-19/Malaysia/3998/2020|EPI ISL 501210|2020-04-01	B.6 (O)	Second wave
hCoV-19/Malaysia/IMR WC9127/2020|EPI ISL 459956|2020-03-30	B.6 (O)	Second wave
hCoV-19/Malaysia/MGI-MAEPS67/2020|EPI ISL 528744|2020-06-04	B.6.2 (O)	Second wave
hCoV-19/Malaysia/MGI-MAEPS54/2020|EPI ISL 528743|2020-06-04	B.6.2 (O)	Second wave
hCoV-19/Malaysia/IMR WC99023/2020|EPI ISL 490099|2020-05-23	B.6.2 (O)	Second wave
hCoV-19/Malaysia/IMR WC81523/2020|EPI ISL 490096|2020-05-23	B.6.2 (O)	Second wave
hCoV-19/Malaysia/IMR WC80558/2020|EPI ISL 490095|2020-05-23	B.6.2 (O)	Second wave
hCoV-19/Malaysia/IMR WC80109/2020|EPI ISL 490094|2020-05-23	B.6.2 (O)	Second wave
hCoV-19/Malaysia/IMR WC81849/2020|EPI ISL 490098|2020-05-23	B.6.2 (O)	Second wave
hCoV-19/Malaysia/IMR WC80066/2020|EPI ISL 490092|2020-05-23	B.6.2 (O)	Second wave
hCoV-19/Malaysia/MGI-MAEPS41/2020|EPI ISL 582124|2020-06-03	B.6.2 (O)	Second wave
hCoV-19/Malaysia/IMR WC99045/2020|EPI ISL 490100|2020-05-23	B.6.2 (O)	Second wave
hCoV-19/Malaysia/IMR WC80031/2020|EPI ISL 490090|2020-05-23	B.6.2 (O)	Second wave
hCoV-19/Malaysia/1399/2020|EPI ISL 501184|2020-03-25	B.6 (O)	Second wave
hCoV-19/Malaysia/8816/2020|EPI ISL 501224|2020-03-18	B.6 (O)	Second wave
hCoV-19/Malaysia/9136/2020|EPI ISL 501225|2020-03-19	B.6 (O)	Second wave
hCoV-19/Malaysia/IMR WC2453/2020|EPI ISL 459954|2020-03-16	B.6.6 (O)	Second wave
hCoV-19/Malaysia/IMR WC12286/2020|EPI ISL 490016|2020-04-10	B.6.6 (O)	Second wave
hCoV-19/Malaysia/5906/2020|EPI ISL 501215|2020-04-06	B.6.6 (O)	Second wave
hCoV-19/Malaysia/2063/2020|EPI ISL 501188|2020-03-26	B.6.6 (O)	Second wave
hCoV-19/Malaysia/2May20-132-Hu/2020|2020-05-02	B.6.6 (O)	Second wave
hCoV-19/Malaysia/188407/2020|EPI ISL 417918|2020-03-18	B.6.6 (O)	Second wave
hCoV-19/Malaysia/IMR WC2423/2020|EPI ISL 459953|2020-03-16	B.6 (O)	Second wave
hCoV-19/Malaysia/9857/2020|EPI ISL 501227|2020-03-21	B.6 (O)	Second wave
hCoV-19/Malaysia/1204/2020|EPI ISL 501183|2020-03-24	B.6 (O)	Second wave
hCoV-19/Malaysia/IMR WC13946/2020|EPI ISL 490047|2020-04-10	B.6 (O)	Second wave
hCoV-19/Malaysia/IIUM316/2020|EPI ISL 455312|2020-04-09	B.6 (O)	Second wave
hCoV-19/Malaysia/7685/2020|EPI ISL 506996|2020-04-10	B.6 (O)	Second wave
hCoV-19/Malaysia/2875/2020|EPI ISL 501198|2020-03-28	B.6 (O)	Second wave
hCoV-19/Malaysia/3133/2020|EPI ISL 501201|2020-03-30	B.6 (O)	Second wave
hCoV-19/Malaysia/1121/2020|EPI ISL 501182|2020-03-24	B.6 (O)	Second wave
hCoV-19/Malaysia/0478/2020|EPI ISL 501178|2020-03-23	B.6 (O)	Second wave

Supplementary Table 2: Genetic markers used for GISAID SARS-CoV-2 clade assignment.

Clade	PANGOLIN Lineages	Genetic markers (WIV04-reference sequence)
S	A	C8782T, T28144C (NS8-L84S)
L	B	C241, C3037, A23403, C8782, G11083, G25563, G26144, T28144, G28882
V	B.2	G11083T (NSP6-L37F), G26144T (NS3-G251V)
G	B.1	C241T, C3037T, A23403G (S-D614G)
GH	B.1*	C241T, C3037T, A23403G (S-D614G), G25563T (NS3-Q57H)
GR	B.1.1	C241T, C3037T, A23403G (S-D614G), G28882A (N-G204R)
GV	B.1	C241T, C3037T, A23403G (S-D614G), C22227T (S-A222V)
O	-	Others

We gratefully acknowledge the following Authors from the Originating laboratories responsible for obtaining the specimens, as well as the Submitting laboratories where the genome data were generated and shared via GISAID, on which this research is based.

All Submitters of data may be contacted directly via www.gisaid.org

Authors are sorted alphabetically.

Accession ID	Originating Laboratory	Submitting Laboratory	Authors
EPI_ISL_410487, EPI_ISL_410488, EPI_ISL_410489	National Public Health Laboratory	National Public Health Laboratory	Yu Kie,C., Norazimah,T., Rehan Shuhada,A.B., Selvanesan,S., Noorliza,M.N. and Hani,M.H.
EPI_ISL_416829	National Public Health Laboratory	Malaysia Genome Institute	Mohd Noor Mat Isa, Irni Suhayu Sapian, Yusuf Muhammad Noor, Nurhezreen Md Iqbal, Mohd Faizal Abu Bakar, Enizza Kasim, Shamsidar Sopie, Siti Noraini Othman, Azrin Ahmad, Nor Azfa Johari, Norazimah Tajudin, Noorliza Mohamad Noordin, W Afiza W Mohd Arifin, Rehan Shuhada Abu Bakar, Yu Kie Chem, Selvanesan Sengol, Hani Mat Hussin, Shahrul Hisham Zainal Ariffin
EPI_ISL_416866, EPI_ISL_416884	National Public Health Laboratory	Malaysia Genome Institute	Mohd Noor Mat Isa, Irni Suhayu Sapian, Yusuf Muhammad Noor, Nurhezreen Md Iqbal, Mohd Faizal Abu Bakar, Enizza Kasim, Shamsidar Sopie, Siti Noraini Othman, Azrin Ahmad, Nor Azfa Johari, Norazimah Tajudin, Noorliza Mohamad Noordin, W Afiza W Mohd Arifin, Rehan Shuhada Abu Bakar, Yu Kie Chem, Selvanesan Sengol, Hani Mat Hussin, Shahrul Hisham Zainal Ariffin
EPI_ISL_416885, EPI_ISL_416886, EPI_ISL_416907	National Public Health Laboratory	Malaysia Genome Institute	Mohd Noor Mat Isa, Irni Suhayu Sapian, Yusuf Muhammad Noor, Nurhezreen Md Iqbal, Mohd Faizal Abu Bakar, Enizza Kasim, Shamsidar Sopie, Siti Noraini Othman, Azrin Ahmad, Nor Azfa Johari, Norazimah Tajudin, Noorliza Mohamad Noordin, W Afiza W Mohd Arifin, Rehan Shuhada Abu Bakar, Yu Kie Chem, Selvanesan Sengol, Hani Mat Hussin, Shahrul Hisham Zainal Ariffin
EPI_ISL_417917	Department of Medical Microbiology, University Malaya Medical Centre	Department of Medical Microbiology	Yoong Min CHONG, Sasheela PONNAMPALAVANAR, Sharifah Faridah SYED OMAR, Adeeba KAMARULZAMAN,Vijayan MUNUSAMY, Chee Kuan WONG, Cindy Shuan Ju TEH, I-Ching SAM, Yoke Fun Chan, University Malaya Medical Centre COVID Team
EPI_ISL_417918	Department of Medical Microbiology, University Malaya Medical Centre	Department of Medical Microbiology, Faculty of Medicine, University of Malaya	Yoong Min CHONG, Sasheela PONNAMPALAVANAR, Sharifah Faridah SYED OMAR, Adeeba KAMARULZAMAN,Vijayan MUNUSAMY, Chee Kuan WONG, Cindy Shuan Ju TEH, I-Ching SAM, Yoke Fun Chan, University Malaya Medical Centre COVID Team
EPI_ISL_417919	Department of Medical Microbiology, University Malaya Medical Centre	Department of Medical Microbiology, Faculty of Medicine, University of Malaya	Yoong Min CHONG, Sasheela PONNAMPALAVANAR, Sharifah Faridah SYED OMAR, Adeeba KAMARULZAMAN,Vijayan MUNUSAMY, Chee Kuan WONG, Fadhil Hadi JAMALUDDIN, Han Ming GAN, Cindy Shuan Ju TEH, I-Ching SAM, Yoke Fun CHAN, University Malaya Medical Centre COVID Team
EPI_ISL_417920	Department of Medical Microbiology, University Malaya Medical Centre	Department of Medical Microbiology, Faculty of Medicine, University of Malaya	Yoong Min CHONG, Sasheela PONNAMPALAVANAR, Sharifah Faridah SYED OMAR, Adeeba KAMARULZAMAN,Vijayan MUNUSAMY, Chee Kuan WONG, Fadhil Hadi JAMALUDDIN, Cindy Shuan Ju TEH, I-Ching SAM, Yoke Fun Chan, University Malaya Medical Centre COVID Team
EPI_ISL_430439	Institute for Medical Research, Infectious Disease Research Centre, National Institutes of Health, Ministry of Health Malaysia	Institute for Medical Research Infectious Disease Research Centre, National Institutes of Health, Ministry of Health Malaysia	Suppiah.J, Mohd-Zawawi.Z, Kalyanasundram.J, Azizan.M-A, Mat-Sharani.S, Hisham.H-A, Tan.L-P, Abdul-Wahid.M-Z, Mohd-Zain.R, Ahmad.N, Thayan.R
EPI_ISL_430440	Institute for Medical Research, Infectious Disease Research Centre, National Institutes of Health, Ministry of Health Malaysia	Institute for Medical Research, Infectious Disease Research Centre, National Institutes of Health, Ministry of Health Malaysia	Suppiah.J, Mohd-Zawawi.Z, Kalyanasundram.J, Azizan.M-A, Mat-Sharani.S, Hisham.H-A, Tan.L-P, Abdul-Wahid.M-Z, Tengku-Abd-Rashid.T-R, Mohd-Zain.R, Ahmad.N, Thayan.R
EPI_ISL_430441, EPI_ISL_430442, EPI_ISL_430443, EPI_ISL_430444	Institute for Medical Research, Infectious Disease Research Centre, National Institutes of Health, Ministry of Health Malaysia	Institute for Medical Research, Infectious Disease Research Centre, National Institutes of Health, Ministry of Health Malaysia	Suppiah.J, Mohd-Zawawi.Z, Kalyanasundram.J, Azizan.M-A, Mat-Sharani.S, Hisham.H-A, Tan.L-P, Abdul-Wahid.M-Z, Tengku-Rogayah.TAR, Mohd-Zain.R, Ahmad.N, Thayan.R
EPI_ISL_455312	Microbiology Unit, Department of Pathology & Laboratory Medicine, IIUM Medical Centre	SEA Microbiome Unit, Faculty of Industrial Sciences & Technology, Universiti Malaysia Pahang	Norhidayah Binti Kamarudin, Ahmad Hafiz Bin Zulkifly, Hajar Fauzan Ahmad, Muhammad Adam Lee Abdullah, Mohd Fazli Farida Asras, Ahmad Mahfuz Gazali, Mohd Nazli Bin Kamarulzaman, IIUM Medical Centre Covid19 Taskforce, UMP Covid19 Team
EPI_ISL_455313	Microbiology Unit, Department of Pathology & Laboratory Medicine, IIUM Medical Centre	SEA Microbiome Unit, Faculty of Industrial Sciences & Technology, Universiti Malaysia Pahang	Hajar Fauzan Ahmad, Norhidayah Kamarudin, Ahmad Hafiz Zulkifly, IIUM Medical Centre Covid19 Taskforce, UMP Covid19 Team
EPI_ISL_455790, EPI_ISL_455791	Institute for Medical Research, Infectious Disease Research Centre, National Institutes of Health, Ministry of Health Malaysia	Malaysia Genome Institute	Mohd Noor Mat Isa, Irni Suhayu Sapian, Yusuf Muhammad Noor, Jeyanthi Suppiah, Nurhezreen Md Iqbal, Enizza Kasim, Zarina Mohd Zawawi, Siti Noraini Othman, Mohd Faizal Abu Bakar, Shamsidar Sopie, Azrin Ahmad, Ravindran Thayan, Norazah Ahmad, Tahir Aris, Shahrul Hisham Zainal Ariffin
EPI_ISL_455792, EPI_ISL_455793	Institute for Medical Research, Infectious Disease Research Centre, National Institutes of Health, Ministry of Health Malaysia	Malaysia Genome Institute	Mohd Noor Mat Isa, Irni Suhayu Sapian, Yusuf Muhammad Noor, Jeyanthi Suppiah, Nurhezreen Md Iqbal, Enizza Kasim, Zarina Mohd Zawawi, Siti Noraini Othman, Mohd Faizal Abu Bakar, Shamsidar Sopie, Azrin Ahmad, Ravindran Thayan, Norazah Ahmad, Tahir Aris, Shahrul Hisham Zainal Ariffin
EPI_ISL_459953	Institute for Medical Research, Infectious Disease Research Centre, National Institutes of Health, Ministry of Health Malaysia	Institute for Medical Research Infectious Disease Research Centre, National Institutes of Health, Ministry of Health Malaysia	Suppiah J, Mohd-Zawawi Z, Kamel KA, Ellan K, Kalyanasundram J, Mohd-Zain R, Thayan R
EPI_ISL_459954, EPI_ISL_459955, EPI_ISL_459956	Institute for Medical Research, Infectious Disease Research Centre, National Institutes of Health, Ministry of Health Malaysia	Institute for Medical Research, Infectious Disease Research Centre, National Institutes of Health, Ministry of Health Malaysia	Suppiah J, Mohd-Zawawi Z, Kamel KA, Ellan K, Kalyanasundram J, Mohd-Zain R, Thayan R
EPI_ISL_459957	Institute for Medical Research, Infectious Disease Research Centre, National Institutes of Health, Minis	Institute for Medical Research, Infectious Disease Research Centre, National Institutes of Health, Minis	Suppiah J, Mohd-Zawawi Z, Kamel KA, Ellan K, Kalyanasundram J, Mohd-Zain R, Thayan R
EPI_ISL_489992, EPI_ISL_489993, EPI_ISL_489994, EPI_ISL_490014, EPI_ISL_490015, EPI_ISL_490016, EPI_ISL_490047, EPI_ISL_490048, EPI_ISL_490089, EPI_ISL_490090, EPI_ISL_490091, EPI_ISL_490092, EPI_ISL_490093, EPI_ISL_490094, EPI_ISL_490095, EPI_ISL_490096, EPI_ISL_490097, EPI_ISL_490098, EPI_ISL_490099, EPI_ISL_490100, EPI_ISL_490101, EPI_ISL_490102, EPI_ISL_490103			
see above	Institute for Medical Research, Infectious Disease Research Centre, National Institutes of Health, Ministry of Health Malaysia	Institute for Medical Research, Infectious Disease Research Centre, National Institutes of Health, Ministry of Health Malaysia	Suppiah J, Mohd-Zawawi Z, Kamel K, Kalyanasundram J, Thayan R
EPI_ISL_501176, EPI_ISL_501177, EPI_ISL_501178, EPI_ISL_501179, EPI_ISL_501180, EPI_ISL_501181, EPI_ISL_501182, EPI_ISL_501183, EPI_ISL_501184, EPI_ISL_501185, EPI_ISL_501186, EPI_ISL_501187, EPI_ISL_501188, EPI_ISL_501189, EPI_ISL_501190, EPI_ISL_501191, EPI_ISL_501192, EPI_ISL_501193, EPI_ISL_501194, EPI_ISL_501195, EPI_ISL_501196, EPI_ISL_501197, EPI_ISL_501198, EPI_ISL_501199, EPI_ISL_501200, EPI_ISL_501201, EPI_ISL_501202, EPI_ISL_501203, EPI_ISL_501204, EPI_ISL_501205, EPI_ISL_501206, EPI_ISL_501207, EPI_ISL_501208, EPI_ISL_501209, EPI_ISL_501210, EPI_ISL_501211, EPI_ISL_501212, EPI_ISL_501213, EPI_ISL_501214, EPI_ISL_501215, EPI_ISL_501216, EPI_ISL_501217, EPI_ISL_501218, EPI_ISL_501219, EPI_ISL_501220, EPI_ISL_501221, EPI_ISL_501222, EPI_ISL_501223, EPI_ISL_501224, EPI_ISL_501225, EPI_ISL_501226, EPI_ISL_501227, EPI_ISL_501228, EPI_ISL_506996, EPI_ISL_506997, EPI_ISL_506998, EPI_ISL_506999, EPI_ISL_507000			
see above	Department of Medical Microbiology, University Malaya Medical Centre	Department of Medical Microbiology, Faculty of Medicine, University of Malaya	Yoong Min CHONG, Jennifer Chong, I-Ching SAM, Yoke Fun CHAN, University Malaya Medical Centre COVID Team
EPI_ISL_528738, EPI_ISL_528739, EPI_ISL_528740, EPI_ISL_528741	Malaysia Genome Institute	Malaysia Genome Institute	Mohd Noor Mat Isa, Irni Suhayu Sapian, Yusuf Muhammad Noor, Nurhezreen Md Iqbal, Mohd Faizal Abu Bakar, Enizza Kasim, Shamsidar Sopie, Siti Noraini Othman, Azrin Ahmad, Nor Azfa Johari, Shahrul Hisham Zainal Ariffin
EPI_ISL_528742	Malaysia Genome Institute	Malaysia Genome Institute	Mohd Noor Mat Isa, Irni Suhayu Sapian, Gan Han Ming, Yusuf Muhammad Noor, Tan Ju Lin, Nurhezreen Md Iqbal, Mohd Faizal Abu Bakar, Enizza Kasim, Shamsidar Sopie, Siti Noraini Othman, Azrin Ahmad, Nor Azfa Johari, Shahrul Hisham Zainal Ariffin
EPI_ISL_528743, EPI_ISL_528744, EPI_ISL_568873, EPI_ISL_568874,	Malaysia Genome Institute	Malaysia Genome Institute	Mohd Noor Mat Isa, Irni Suhayu Sapian, Yusuf Muhammad Noor, Nurhezreen Md Iqbal, Mohd Faizal Abu Bakar, Enizza Kasim, Shamsidar Sopie, Siti Noraini Othman, Azrin Ahmad, Nor Azfa Johari, Shahrul Hisham Zainal Ariffin

EPI_ISL_568875, EPI_ISL_582124
